# Adjuvant ICIs Plus Targeted Therapies Reduce HCC Recurrence after Hepatectomy in Patients with High Risk of Recurrence

**DOI:** 10.3390/curroncol30020132

**Published:** 2023-01-31

**Authors:** Jianming Yang, Shijie Jiang, Yewu Chen, Jian Zhang, Yinan Deng

**Affiliations:** Department of Hepatic Surgery, The Third Affiliated Hospital of Sun Yat-sen University, Guangzhou 510630, China

**Keywords:** hepatocellular carcinoma, immune checkpoint inhibitors, hepatectomy, targeted therapies, recurrence

## Abstract

Background: The high recurrence rate of hepatocellular carcinoma (HCC) after hepatectomy usually results in poor prognosis. To the best of our knowledge, no study has reported the efficacy of immune checkpoint inhibitors (ICIs) plus targeted therapies on preventing HCC recurrence after hepatectomy. Thus, the aim of this study was to investigate the benefits and safety of applying adjuvant ICIs plus targeted therapies after hepatectomy for patients at high risk of HCC recurrence. Methods: A total of 196 patients with any risk factors for recurrence who underwent hepatectomy for HCC were reviewed in this retrospective study. Results: Compared with the control group (n = 158), ICIs plus targeted therapies (n = 38) had a significantly higher recurrence-free survival (RFS) rate in univariate analysis (HR, 0.46; 95% confidence interval [CI], 0.24–0.90; *p* = 0.020), multivariate analysis (adjusted HR, 0.62; 95%CI, 0.49–0.79; *p* < 0.001) and propensity score-matched analysis (HR, 0.35; 95%CI, 0.16–0.75; *p* = 0.005). Subgroup analyses also showed that postoperative adjuvant ICIs plus targeted therapies might reduce HCC recurrence in patients with the most of risk factors. Conclusion: Postoperative adjuvant ICI plus targeted therapies may reduces early HCC recurrence in patients with a high risk of recurrence, and the treatments are well tolerated.

## 1. Introduction

Hepatectomy is one of the most effective treatments for eradicating hepatocellular carcinoma (HCC). However, according to recent reports, the 5-year HCC recurrence or metastasis rate after hepatectomy is as high as 70–80% [1,2,3], resulting in poor outcomes. Since the number of tumor lesions and size, high preoperative alpha-fetoprotein (AFP) level, microvascular invasion (MVI), macrovascular invasion, macrovascular tumor thrombus, poor tumor grade and incomplete tumor capsules are widely accepted as independent risk factors associated with early recurrence of HCC after resection [4,5,6,7], preventive intervention for these patients is necessary.

Great efforts have been made to explore effective preventive interventions to reduce HCC recurrence after hepatectomy. In recent years, transarterial chemoembolization (TACE) [8,9,10,11,12] and hepatic artery infusion chemotherapy (HAIC) [13,14,15,16] are gaining increasing interest as minimally invasive adjuvant therapies to prevent HCC recurrence after hepatectomy, especially for patients with a high risk of recurrence. Three meta-analyses have suggested that adjuvant TACE might reduce the intrahepatic HCC recurrence in patients with a tumor diameter > 5 cm, MVI, or multiple lesions [9,10,11,12]. Several retrospective studies have reported that patients with macro- or micro-vascular invasions might benefit from adjuvant HAIC [14,15,16]. However, since TACE and HAIC are both locoregional therapies, their anti-tumor effects mainly focus on curbing intrahepatic recurrence, but not extrahepatic metastasis. To date, it remains controversial that postoperative adjuvant targeted therapies could benefit patients with HCC [17,18,19,20]. Though the STORM trial, the only randomized controlled trial (RCT) that investigated the efficacy of Sorafenib as an postoperative adjuvant therapy, suggested that patients might not benefit from adjuvant Sorafenib [17], other retrospective studies demonstrated that Sorafenib would decrease the recurrence rate after surgery, especially in patients at high risk of recurrence with MVI, poor tumor grade, or tumor at stage BCLC-C [18,19,20].

Present studies have shown that immune checkpoint inhibitors (ICIs) therapies are novel and effective regimens for intermediate–advanced HCC [21,22,23], and since the success of IMbrave-150 [22], the combination of ICI and targeted therapy has been recommended and widely applied as a first-line treatment for intermediate–advanced HCC. Hence, choosing ICI plus targeted therapy as systemic adjuvant therapy for preventing HCC recurrence after resection might be feasible and promising. Several RCTs assessing postoperative adjuvant ICIs-based treatment are ongoing. However, to the best of our knowledge, no preliminary conclusions have been drawn from these RCTs or other retrospective studies. Thus, we conducted this retrospective cohort study to explore the benefit of adjuvant ICIs plus targeted therapies after hepatectomy in patents with HCC with any risk factors of recurrence.

## 2. Methods

### 2.1. Study Population

**Figure 1:**
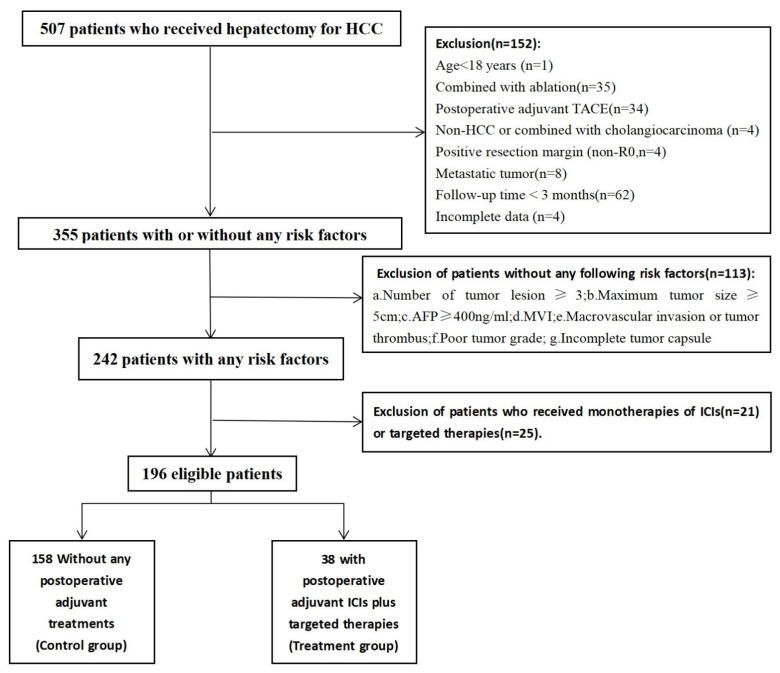
Patients flow diagrams. HCC = Hepatocellular carcinoma; TACE = Transarterial chemoembolization; AFP = Alpha-fetoprotein; MVI = Microvascular invasion; ICIs = Immune Checkpoint Inhibitors.

Our study was approved by the institutional review boards of the Third Affiliated Hospital of Sun Yat-sen University (Number: [2022]02-333-01). Due to the retrospective nature of this study, the need for informed consent was therefore waived, and all the clinical data were collected and reviewed confidentially from the hospital electronic database. A total of 507 consecutive patients who received hepatectomy for HCC, from January 2020 to April 2022, at The Third Affiliated Hospital of Sun-Yet Sen University (Guangzhou, China) were enrolled in this retrospective study. The following exclusion criteria were adopted (Figure 1): age < 18 years (n = 1); hepatectomy combined with ablation (n = 35); postoperative adjuvant TACE (n = 34); pathologically confirmed non-HCC or combined with cholangiocarcinoma (n = 4); pathologically confirmed positive resection margin (non-R0 resection, n = 4); preoperative benign lesions confirmed as metastatic tumors after surgery (n = 8); follow-up time < 3 months without any events(n = 62); incomplete data (n = 4); lack of risk factors (n = 113); received monotherapies of ICIs or targeted therapies (n = 46). The risk factors for HCC recurrence included number of tumor lesions ≥ 3, maximum tumor size ≥5 cm, preoperative AFP ≥ 400 ng/mL, MVI, macrovascular invasion or tumor thrombus, poor tumor grade (Edmondson–Steiner grade III/IV), and incomplete tumor capsules; the final population of 196 patients was analyzed.

### 2.2. Treatments

All patients were treated with radical (R0) hepatectomy, and they were stratified into two groups according to postoperative adjuvant treatments: non-adjuvant treatment (control group, n = 158) and ICIs plus targeted therapies (treatment group, n = 38). ICIs treatments included atezolizumab (1200 mg, q3w, IV drip), camrelizumab (200 mg, q3w, IV drip), sintilimab (200 mg, q3w, IV drip) and tislelizumab (200 mg, q3w, IV drip). Targeted therapy treatments included sorafenib (400 mg, bid, oral), apatinib (250 mg, qd, oral), lenvatinib (<60 kg, 8 mg; ≥60 kg, 12 mg, qd, oral), bevacizumab (15 mg/kg, q3w, IV drip) and anlotinib (12 mg, qd, oral). The regimens of adjuvant ICIs and targeted therapies used are shown in Table 1. Out of the 11 regimens administered in total, four have passed the phase II or III clinical trials [22,24,25,26], and the efficacy and safety of five regimens have been reported in retrospective studies [27,28,29,30,31]. Two tislelizumab-based regimens have not been reported regarding HCC. However, the use of tislelizumab, an ICI that passed the phase III trial for HCC, have been reported in combination with apatinib or lenvatinib for other cancers such as gastric cancer and gallbladder cancer, and the efficacy and safety of these therapies were promising [32,33]. In addition, a phase II trial evaluating efficacy of tislelizumab plus lenvatinib is ongoing (NCT04401800). Adjuvant ICIs plus targeted therapies were initiated within two months (average time, 29d; in the range 7–57d) following hepatectomy after obtaining written informed consent from patients who were followed up closely for any adverse events. All adjuvant treatments were reduced or discontinued once any poor drug-related adverse events occurred.

### 2.3. Outcomes and Follow-Up Assessment

The outcome was HCC recurrence confirmed by contrast-enhanced computed tomography (CT), ultrasonography or enhanced magnetic resonance imaging (MRI). The index date was defined as the date of hepatectomy for HCC. All patients’ recurrence-free survival (RFS) was computed from the index date to the date of confirmation of recurrence, or the last follow-up date (August 2022). During the follow-up period, patients were advised to undergo evaluation including ultrasonography, contrast-enhanced CT, liver function tests and serum alpha-fetoprotein (AFP). When extrahepatic metastasis was suspected, positron emission tomography-computed tomography (PET-CT) was conducted.

### 2.4. Statistical Methods 

Continuous data were expressed as median (range) and compared using *t*-test; categorical data, which were expressed as exact numbers and proportions, were compared using chi-square and Fisher’s tests. The Kaplan–Meier method and log-rank test were used to compare RFS between groups. Cox proportional risk regression model was applied for multivariate analyses, and variables having a *p* < 0.10 in univariate analysis were eligible for the Cox regression models.

To further avoid selection bias and potential confounding, we performed a propensity score-matched (PSM) analysis. A 1:1 nearest neighbor matching scheme with a caliper size of 0.2 was used to identify the final PSM cohort; propensity scores were computed using the following 14 variables: sex, age, Eastern Cooperative Oncology Group performance status (ECOG-PS) score, tumor grade, capsular invasion, AFP, preoperative locoregional treatments (including TACE, radiofrequency ablation and portal vein embolization), macrovascular tumor thrombus, number of lesion, maximum tumor size, cirrhosis (confirmed by CT or ultrasound), HBsAg, MVI and macrovascular invasion. *p* < 0.05 was considered to be statistically significant. All statistical analyses were performed using R statistical software, version 4.1.0 (R foundation Inc.; http://cran.r-project.org/, accessed on 21 September 2022). R packages including MatchIt, survival, survminer, tableone, and ggplot2 were used to analyze the statistics and create the figures and tables. 

## 3. Results

### 3.1. Baseline Characteristics and Follow-Up Status

The baseline characteristics of the control and treatment groups are presented in Table 2. Among the entire cohort of 196 patients, most were males (n = 172, 87.7%) and had tested positive for serum HBsAg (n = 175, 89.3%), with a median age of 54 (IQR, 46–62). Most of the variables had no significant difference between the two groups in the entire cohort, except that the control group was older than the treatment group (55.0 y vs. 50.5 y, *p* = 0.026), and the treatment group had a poorer BCLC-stage (65.8% vs. 31.0%, *p* < 0.001), more tumor lesions (31.6% vs. 15.8%, *p* = 0.046), more macrovascular invasions (63.2% vs. 29.1%, *p* < 0.001), and received more preoperative locoregional therapies (34.2% vs. 13.9%, *p* = 0.007; resulted in a smaller tumor size, 3.6 cm vs. 4.5 cm, *p* = 0.048) than the control group, which were balanced in the PSM cohort. In the treatment group, most patients had two or more risk factors of recurrence simultaneously (n = 30, 79.0%); the most common reasons for using ICI plus targeted therapy were macrovascular invasion (n = 24, 63.2%) and large tumor size (≥5 cm; n = 24, 63.2%). The median follow-up time of the entire cohort was 365.4 d, with 363.9 d in the control group and 371.2 d in the treatment group. By the cut-off date, 5 (2.5%) patients had died due to intraperitoneal hemorrhage, infection, or liver failure, and 84 (42.8%) had experienced HCC recurrence with a median recurrence time of 214 d.

### 3.2. Survival Analysis of Postoperative Adjuvant Treatments in the Overall and Propensity-Matched Cohorts

**Figure 2:**
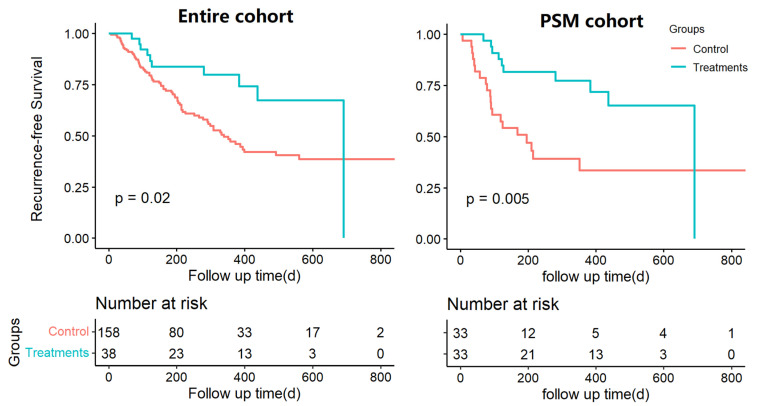
Comparison of recurrence-free survival curve between control and treatment group for HCC recurrence in the entire cohort and propensity score-matched (PSM) cohort.

In both the entire and PSM cohorts, the treatment group showed a significantly better RFS than the control group (for the entire cohort: hazard ratio [HR], 0.46; 95% confidence interval [CI], 0.24–0.90; *p* = 0.020; for the PSM cohort: HR, 0.35; 95%CI, 0.16–0.75, *p* = 0.005; Figure 2) and a longer median RFS time (for the entire cohort:22 vs. 11 months; for the PSM cohort:6 vs. 22 months). In the multivariable Cox regression analysis, adjuvant ICIs plus targeted therapies were significantly associated with a lower risk of HCC recurrence after hepatectomy (adjusted HR [aHR], 0.62; 95%CI, 0.49–0.79, *p* < 0.001; Table 3). Other independent factors associated with HCC recurrence were MVI (aHR, 2.04; 95%CI, 1.31–3.15, *p* = 0.001), macrovascular invasion (aHR, 2.21; 95%CI, 1.39–3.52, *p* < 0.001), number of lesions ≥ 3 (aHR, 1.96; 95%CI, 1.14–3.36, *p* = 0.015) and cirrhosis (aHR, 1.69; 95%CI, 1.06–2.70, *p* = 0.026).

### 3.3. Exploratory Subgroup Analyses of Postoperative Adjuvant ICIs Plus Targeted Therapies

**Figure 3:**
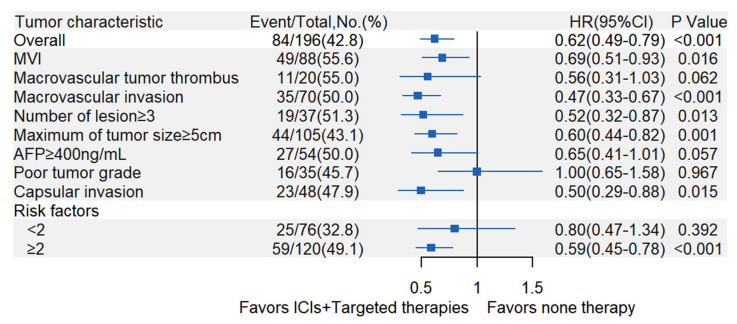
Forest plot for subgroup analysis of the comparison between control and treatment groups. MVI = Microvascular invasion; AFP = Alpha-fetoprotein.

As presented in Figure 3, patients were stratified into several subgroups according to the risk factors of HCC recurrence, and then multivariable stratified analysis was performed to explore the benefit of adjuvant ICIs plus targeted therapies after hepatectomy in different subgroups. The effect of preventing HCC recurrence was mostly observed in patients with the following tumor characteristics: number of tumor lesions ≥3 (aHR, 0.52; 95%CI, 0.32–0.87; *p* = 0.013), maximum tumor size ≥ 5 cm (aHR, 0.60; 95%CI, 0.44–0.82; *p* = 0.001), MVI (aHR, 0.69; 95%CI, 0.51–0.93; *p* = 0.016), macrovascular invasion (aHR, 0.47; 95%CI, 0.33–0.67; *p* < 0.001) and incomplete capsules (aHR, 0.50; 95%CI, 0.29–0.88; *p* = 0.015). Moreover, we found that a similar effect appeared in those with two or more simultaneous risk factors (aHR, 0.59; 95%CI, 0.45–0.78; *p* < 0.001), which was not observed in patients with only one risk factors (aHR, 0.80; 95%CI, 0.47–1.37; *p* = 0.392).

### 3.4. Safety of Postoperative Adjuvant Therapies

The adverse events associated with adjuvant ICIs plus targeted therapies are summarized in Table 4. Overall, adjuvant ICIs plus targeted therapies was safe and well tolerated, with four (10.5%) grade 1 or 2 adverse events and one (2.6%) grade 3 adverse event. All of the adverse events were immune-related adverse events (irAEs), which could be controlled after corticosteroid therapy.

## 4. Discussion

Hepatectomy is the primary curative treatment patients with HCC with good liver function reserves. However, the high HCC recurrence rate (up to 70–80% at 5 years after surgery) remains an important clinical problem that significantly reduces the efficacy of hepatectomy. Recent studies have shown that ICIs-based therapies are effective regimens for intermediate–advanced HCC [21,22,23], suggesting that ICIs-based therapy could be considered a promising postoperative adjuvant treatment in improving outcomes after hepatectomy. Though several clinical trials evaluating postoperative adjuvant ICIs-based therapies are ongoing, there are no preliminary conclusions yet from these RCT studies.

From this retrospective study, to the best of our knowledge, we were the first to report the potential benefits and feasibility of postoperative adjuvant ICIs plus targeted therapies in patients presenting with risk factors of HCC recurrence. We found that the group receiving adjuvant ICIs plus targeted therapies was associated with a lower risk of early HCC recurrence compared with the control group in univariate and multivariate analysis. Before PSM, the treatment group had a more advanced BCLC-stage, more tumor lesions, and more macrovascular invasions, and received more preoperative locoregional therapies. This could be explained by the reason that the adjuvant ICI plus targeted therapy was more likely to be applied in patients with higher risk of HCC recurrence, and these differences were balanced in the PSM cohort, which again showed a benefit of adjuvant ICIs plus targeted therapy. Moreover, the subgroup analyses were conducted according to clinicopathological characteristics, including all risk factors considered as selective criteria in this study. We noted that the benefit of postoperative adjuvant ICIs plus targeted therapies could be observed in most of the subgroups. Interestingly, the result was positive in the group of two or more risk factors, but not in the group of only one risk factor, which indicated that the higher the risk of HCC recurrence, the greater the benefit from these therapies.

Compared with late recurrence, early recurrence mainly originates from intrahepatic metastasis and is driven by aggressive characteristics of the primary tumor such as tumor size, multiple tumor lesions, vascular invasion or higher serum AFP level [4,5,6,7,34]. In addition to tumor characteristics, immune mechanisms have also been shown to be associated with HCC recurrence following hepatectomy [35,36,37,38]. In particular, PD-L1/PD-1 and VEGF pathways are both believed to play a critical and synergistic role in tumor immune evasion [39,40]. On the basis of the evidence above and findings from our study, we postulated that ICIs plus targeted therapies might reduce HCC recurrence by inhibiting PD-L1/PD1 and VEGF, which mediate the immune system to treat microscopic pre-existing tumors or intrahepatic metastases, and inhibit tumor immune escape.

The safety of ICI-based therapies is always a matter of concern. In our study, only five (13.1%) patients experienced AEs during the follow-up, which indicated that postoperative adjuvant ICIs plus targeted therapy was well tolerated. However, this result was not consistent with the results from the present clinical trials [22,24,25,26]. All the data regarding AEs were obtained from outpatient records that were lacking details of AEs from targeted therapies, such as hand-foot syndrome, hypertension and diarrhea, as they are more likely to be ignored in outpatient compared with irAEs. Therefore, the probability of AEs may be underestimated and needs to be evaluated in further clinical trials. Another concern is the safety of liver transplantation (LT) for tumor progression or liver failure following adjuvant ICI-based treatment after hepatectomy. Theoretically, the allograft rejection rate is supposed to increase in patients receiving ICI therapy before LT. While early death from liver failure after LT for patients who received pre-LT ICI treatments has been described in previous case reports, another case-series reported successful transplants after ICI use, with only 2 patients (out of 18) undergoing mild rejection, which was successfully treated with the adjustment of their immunosuppression regimens [41]. Thus, this remains controversial, and further study is mandatory to explore the safety of LT after ICI therapy.

In our study, eight risk factors were used to select appropriate patients; nevertheless, based on the consideration of efficacy and safety, a more rigorous screening procedure should be performed before use of adjuvant therapy. Recently, machine learning has been actively used in the development and validation of predictive models. Verma et.al. [42] described a quantitative systems pharmacology models (QSP) on the basis of a machine learning approach, which successfully identified biomarkers that could facilitate patient selection and ultimately improve the success of ICI treatment. Using a similar model combined with other HCC resection models [43,44] could provide assistance for patient screening, and selection of therapeutic regimens, that would improve the efficacy and safety of adjuvant ICI plus targeted therapy.

The major limitation of our study is that as a retrospective study based on a single-center observational data with a small sample size, our study may be subject to selection bias and confounding. Indeed, the high cost and unclear treatment efficacy of ICIs plus targeted therapies made it quite difficult to enlarge the sample size. In addition, the short follow-up period might have missed recurrence that was supposed to happen, which would lead to bias. A longer follow-up time in future studies is mandatory. Furthermore, the regimens for adjuvant therapies were not unified (Table 1). Although most studies have testified the efficacy of these regimens on intermediate–advanced HCC, the heterogeneity of clinical efficacy between different regimens is expected and unavoidable, though it results in analysis bias. Finally, given that those who underwent HCC recurrence or metastasis will receive different treatments, including secondary hepatectomy, TACE, ablation, or ICIs-based therapies, we did not consider overall survival (OS) as one of the endpoints of our study.

## 5. Conclusions

Our study showed that postoperative adjuvant ICI plus targeted therapies might decrease early HCC recurrence in patients a with high risk of recurrence, and the treatment was feasible and well tolerated. Multicenter clinical trials on a larger scale are mandatory to validate our results.

## Figures and Tables

**Table 1:** Combination regimens of postoperative adjuvant ICIs plus targeted therapies.

Adjuvant ICIs	Targeted Therapies	Entire Cohort (n = 38)	PSM Cohort (n = 33)
Sintilimab	Anlotinib [24]	9	9
	Apatinib [25]	7	6
	Lenvatinib [27]	4	3
	Sorafenib [31]	1	1
Camrelizumab	Anlotinib [30]	3	3
	Apatinib [26]	7	6
	Lenvatinib [28]	2	2
	Sorafenib [29]	1	1
Tislelizumab	Apatinib [32]	1	1
	Lenvatinib [33]	2	0
Atezolizumab	Bevacizumab [22]	1	1

**Table 2:** Baseline characteristics of control and treatment group in entire cohort and propensity-score-matched (PSM) cohort.

	Entire Cohort (n = 196)			PSM Cohort (n = 66)		
Characteristic	Adjuvant ICIs Plus Targeted Therapies		*p*	Adjuvant ICIs Plus Targeted Therapies		*p*
	No (n = 158)	Yes (n = 38)		No (n = 33)	Yes (n = 33)	
Gender (%)			0.933			>0.99
female	20 (12.7)	4 (10.5)		3 (9.1)	3 (9.1)	
male	138 (87.3)	34 (89.5)		30 (90.9)	30 (90.9)	
Age (y, median [IQR])	55.0 [47.0, 63.0]	50.5 [44.0, 54.8]	0.026	49.0 [44.0, 56.0]	50.0 [44.0, 55.0]	0.847
Diabetes (%)	18 (11.4)	7 (18.4)	0.371	4 (12.1)	7 (21.2)	0.509
Hypertention (%)	30 (19.0)	6 (15.8)	0.823	7 (21.2)	6 (18.2)	>0.99
HbsAg (%)			0.739			>0.99
positive	140 (88.6)	35 (92.1)		32 (97.0)	31 (93.9)	
negative	18 (11.4)	3 (7.9)		1 (3.0)	2 (6.1)	
Cirrhosis (%)	82 (51.9)	26 (68.4)	0.098	18 (54.5)	22 (66.7)	0.450
Child-Pugh grade (%)			0.459			0.063
A	146 (92.4)	37 (97.4)		28 (84.8)	33 (100.0)	
B	12 (7.6)	1 (2.6)		5 (15.2)	0 (0.0)	
BCLC-stage (%)			<0.001			0.469
A	73 (46.2)	4 (10.5)		8 (24.2)	4 (12.1)	
B	12 (7.6)	6 (15.8)		3 (9.1)	6 (18.2)	
C	49 (31.0)	25 (65.8)		20 (60.6)	20 (60.6)	
Preoperative locoregional therapies(%)	22 (13.9)	13 (34.2)	0.007	9 (27.3)	9 (27.3)	>0.99
ECOG score ≥1 (%)	9 (5.7)	3 (7.9)	0.896	1 (3.0)	3 (9.1)	0.606
Maximum tumor size(cm, median [IQR])	4.5 [2.6, 6.6]	3.6 [1.1, 5.7]	0.048	5.5 [2.7, 8.1]	3.8 [1.7, 5.7]	0.068
Number of lesion (%)			0.046			>0.99
<3	133 (84.2)	26 (68.4)		25 (75.8)	25 (75.8)	
≥3	25 (15.8)	12 (31.6)		8 (24.2)	8 (24.2)	
Macrovascular invasion (%)	46 (29.1)	24 (63.2)	<0.001	21 (63.6)	19 (57.6)	0.801
Macrovascular tumor thrombus (%)	15 (9.5)	5 (13.2)	0.710	5 (15.2)	4 (12.1)	>0.99
AFP (ng/mL, median [IQR])	39.6 [4.8, 599.0]	11.3 [4.1, 169.7]	0.177	45.9 [6.4, 487.0]	12.6 [4.1, 293.1]	0.145
Poor tumor grade (%)	27 (17.1)	8 (21.1)	0.736	7 (21.2)	6 (18.2)	>0.99
Incomplete Capsule (%)	40 (25.3)	8 (21.1)	0.735	7 (21.2)	8 (24.2)	>0.99
MVI (%)	72 (45.6)	16 (42.1)	0.838	18 (54.5)	16 (48.5)	0.805
Location of recurrence			0.138			0.043
intrahepatic	56 (35.4)	7 (18.4)		17 (51.5)	7 (21.2)	
extrahepatic	4 (2.5)	1 (2.6)		0 (0.0)	1 (3.0)	
both	14 (8.9)	2 (5.3)		3 (9.1)	2 (6.1)	
Follow up time (d, mean (SD))	363.9 (231.6)	371.2 (189.7)	0.858	331.6 (233.7)	394.2 (189.2)	0.237

**Table 3:** Univariate and multivariate analyses for HCC recurrence after hepatectomy in the entire cohort.

Characteristics	Univariate			Multivariate		
	HR	95%CI	*p*	aHR	95%CI	*p*
Gender, male/female	0.75	0.40–1.37	0.340			
Age, >60 y/<60 y	0.95	0.59–1.51	0.817			
Hypertention, yes/no	1.07	0.63–1.82	0.815			
Diabetes, yes/no	0.80	0.40–1.60	0.523			
HBV infection, yes/no	2.34	0.95–5.78	0.058	2.17	0.86–5.46	0.097
**ICIs plus targeted therapies, yes/no**	**0.46**	**0.24–0.90**	**0.020**	**0.62**	**0.49–0.79**	**<0.001**
Preoperative locoregional therapies, yes/no	1.44	0.84–2.45	0.180			
ECOG score, 1–2/0 point	1.47	0.68–3.20	0.325			
Child-Pugh grade, B/A	1.43	0.66–3.09	0.366			
**MVI, yes/no**	**2.24**	**1.45–3.46**	**<0.001**	**2.04**	**1.31–3.15**	**0.001**
Tumor grade, poor/well-moderate	1.15	0.67–1.98	0.618			
Capsular invasion, yes/no	1.17	0.72–1.89	0.523			
AFP, ≥400/<400 ng/ml	1.32	0.84–2.09	0.231			
**Macrovascular invasion, yes/no**	**1.49**	**0.97–2.3**	**0.069**	**2.21**	**1.39–3.52**	**<0.001**
Macrovascular tumor thrombus, yes/no	1.59	0.84–3.01	0.147			
**Number of lesion, ≥3/<3**	**1.72**	**1.02–2.88**	**0.038**	**1.96**	**1.14–3.36**	**0.014**
Maximum tumor diameter, ≥5/<5 cm	1.15	0.75–1.77	0.514			
**Cirrhosis, yes/no**	**1.72**	**1.10–2.68**	**0.016**	**1.69**	**1.06–2.70**	**0.026**

**Table 4:** Adverse events (AEs) of ICIs plus targeted therapies.

AEs	Grade1 (%)	Grade2 (%)	Grade3/4 (%)
Hepatitis	0	1 (2.6)	0
Thyroiditis	0	1 (2.6)	0
Dermatitis	0	2 (5.3)	0
Hypopituitarism	0	0	0
Gastroenteritis	0	0	1 (2.6)
Pancreatitis	0	0	0
Pneumonia	0	0	0
Myocarditis	0	0	0

## Data Availability

The data that generate the results of this study are available on request from the corresponding author: Yinan Deng, Department of Hepatic Surgery, The Third Affiliated Hospital of Sun Yat-sen University, Guangzhou, China.
